# Bioactive strawberry fruit (*Arbutus unedo* L.) extract remedies paraquat-induced neurotoxicity in the offspring prenatally exposed rats

**DOI:** 10.3389/fnins.2023.1244603

**Published:** 2023-10-12

**Authors:** Zakaria Ait Lhaj, Hind Ibork, Sara El Idrissi, Farida Ait Lhaj, Mansour Sobeh, Wael M. Y. Mohamed, Meryem Alamy, Khalid Taghzouti, Oualid Abboussi

**Affiliations:** ^1^Physiology and Physiopathology Team, Faculty of Sciences, Genomic of Human Pathologies Research Centre, Mohammed V University, Rabat, Morocco; ^2^Laboratory of Nanomaterials, Nanotechnologies and Environment, Faculty of Sciences, Center of Materials, Mohammed V University, Rabat, Morocco; ^3^AgroBiosciences Research Division, Mohammed VI Polytechnic University, Ben-Guerir, Morocco; ^4^Basic Medical Science Department, Kulliyyah of Medicine, International Islamic University Malaysia, Kuantan, Pahang, Malaysia

**Keywords:** *Arbutus unedo* L., paraquat, dopamine, redox balance, neurotoxicity

## Abstract

**Background:**

Paraquat (1,1′-dimethyl-4-4′-bipyridinium dichloride) exposure is well-established as a neurotoxic agent capable of causing neurological deficits in offspring. This study aimed to investigate therapeutic effects of *Arbutus unedo* L. aqueous extract (AU) against paraquat (PQ) exposure.

**Methods:**

For that the phytoconstituents of AU was determined by LC/MS, and then its antioxidant potential was assessed by DPPH and ABTS assays. The assessment included its impact on cell viability and mitochondrial metabolism using N27 dopaminergic cells. Additionally, we evaluated the effects of prenatal PQ exposure on motor coordination, dopamine levels, trace element levels, and total antioxidant capacity (TAC) in rat progeny.

**Results:**

The phytochemical profile of AU extract revealed the presence of 35 compounds, primarily phenolic and organic acids, and flavonoids. This accounted for its strong *in vitro* antioxidant activities against DPPH and ABTS radicals, surpassing the activities of vitamin C. Our findings demonstrated that AU effectively inhibited PQ-induced loss of N27 rat dopaminergic neural cells and significantly enhanced their mitochondrial respiration. Furthermore, daily post-treatment with AU during the 21 days of the rat's pregnancy alleviated PQ-induced motor deficits and akinesia in rat progeny. These effects inhibited dopamine depletion and reduced iron levels in the striatal tissues. The observed outcomes appeared to be mediated by the robust antioxidant activity of AU, effectively counteracting the PQ-induced decrease in TAC in the blood plasma of rat progeny. These effects could be attributed to the bioactive compounds present in AU, including phenolic acids such as gallic acid and flavonoids such as quercetin, rutin, apigenin, glucuronide, and kaempferol, all known for their potent antioxidant capacity.

**Discussion:**

In conclusion, this preclinical study provided the first evidence of the therapeutic potential of AU extract against PQ-induced neurotoxicity. These findings emphasize the need for further exploration of the clinical applicability of AU in mitigating neurotoxin-induced brain damage.

## 1. Introduction

Paraquat (PQ) is extensively employed as an herbicide in agricultural practices and has been found to pose detrimental effects on dopaminergic neurons. These effects are primarily attributed to oxidative stress-mediated damage inflicted on lipids, proteins, DNA, and RNA. The process involves the generation of reactive oxygen species (ROS), including superoxide radicals, hydroxyl radicals, and hydrogen peroxide (Colle and Farina, 2021). Notably, PQ-induced neurotoxicity operates *via* a distinct mechanism compared to that of 1-methyl-4-phenyl-1,2,3,6-tetrahydropyridine (MPTP). PQ generates ROS within cells through redox cycling, while MPPT-induced neurodegeneration encompasses additional mechanisms (Choukairi et al., 2015). PQ-induced oxidative stress can have detrimental effects on mitochondria, leading to damage and dysfunction. This impairs the ability of dopaminergic neurons to produce adenosine triphosphate (ATP) and meet their metabolic demands (Somayajulu-Nitu et al., 2009; Chaouhan et al., 2022). Consequently, mitochondrial dysfunction and energy deficits play a significant role in the degeneration of dopaminergic neurons and the manifestation of Parkinson's disease (PD)-like behavior (Moradi Vastegani et al., 2023). Moreover, PD prominently affects striatal dopamine, leading to motor impairments due to the loss of dopaminergic neurons in the substantia nigra pars compacta (SNpc) (Gerfen, 2000; Smith and Villalba, 2008; Lima, 2013; Huh et al., 2022). Enzymes such as tyrosine hydroxylase (TH) and aromatic L-amino acid decarboxylase (L-DOPA) are involved in dopamine metabolism as their distribution contributes to dopamine level imbalances and motor dysfunction (Lipski et al., 2011; Alrashidi, 2022). Preclinical studies have shown that PQ administration leads to reduced motor activity and a dose-dependent loss of striatal TH-positive neurons (Colle and Farina, 2021; Sun et al., 2023). In addition to dopamine dysregulation, trace element levels in the striatum play a crucial role in neurological diseases such as PD, for example, an imbalance in iron, copper, zinc, and manganese homeostasis can lead to oxidative stress and neurotoxicity (Fukushima et al., 2011; Stelmashook et al., 2014; Bjorklund et al., 2018; Vuuren et al., 2020).

In addition to the well-documented detrimental effects of PQ exposure, there is evidence of prenatal exposure to PQ as it readily crosses the placenta (Berry et al., 2010). This may increase the risk of developmental or post-developmental effects in rodent offspring, including neurobehavioral changes, alterations in dopamine metabolism, and cognitive dysfunction (Chakrabarti, 2015; Ait-Bali et al., 2016). The detrimental effects of PQ on dopaminergic neurons, striatal dopamine metabolism, mitochondrial function, and trace element dysregulation in neurological disorders highlight the need for therapeutic interventions. In this respect, antioxidant compounds, selenium compounds (e.g., ebselen or diphenyl diselenide), and metal chelators are commonly utilized to treat these disorders. However, some of them displayed negative side effects. On the other hand, plant extracts and their phytochemical constituents are highly effective in the treatment of several neurological conditions (Aruoma et al., 2003; Sapkota et al., 2010; Beppe et al., 2014; Solayman et al., 2016). *Arbutus unedo* L. (*A. unedo*), also known as a strawberry tree, belongs to the Ericaceae family, and it is widely distributed in the Mediterranean region (Carral, 2015). In Morocco, *A. unedo* is growing wild in different bioclimatic zones and is used in traditional medicine. It was recently reported that *A. unedo* fruits provided a rich nutritional value and contained health-promoting components providing almost 42% of the recommended daily allowance (RDA) for fiber and 36% for zinc as well as iron and manganese (Ait Lhaj et al., 2021a). Alongside this higher nutritional potential, these fruits are of great scientific interest due to their several pharmacological effects, such as depurative, hemostatic, anti-inflammatory, antioxidant, antitumor, and neuroprotective (Fortalezas et al., 2010; Šic Žlabur et al., 2020; Tenuta et al., 2020). However, there are no deep scientific studies about the effect of this fruit against neurodegenerative diseases, except the study suggests the potential effect of *A. unedo* extract fruit against neurodegenerative diseases due to the presence of several flavonoids and phenolic acids (Nunes and Carvalho, 2017). In this context, the study aims to investigate the therapeutic effects of *A. unedo* extract (AU) in the context of PQ-induced neurotoxicity. The investigation includes studying its effects on dopaminergic neuronal cells as well as in offspring prenatally exposed to PQ. By examining the effects of AU on dopamine levels, dopamine metabolism, striatal trace element levels, and mitochondrial function, this study will provide valuable insights into the potential mechanisms underlying its therapeutic actions. Understanding the interplay between AU extract, striatal dopamine, mitochondrial metabolism, and trace element homeostasis may contribute to the development of novel therapeutic strategies for neurodegenerative disorders.

## 2. Materials and methods

### 2.1. Plant material and preparation procedure

Fruits of the wild *A. unedo* were harvested from the northwest of Morocco (Chefchaouen region), after identification by Pr. Hamid Khamar at the National Scientific Institute of Rabat (Department of Botany and Plant Ecology); a voucher specimen was deposited in the National Herbarium of Scientific Institute of Rabat and registered under the number code RAB111500. The fruits were lyophilized, then ground to obtain a fine powder, and stored in a deep freezer at −20°C for subsequent analyses. The aqueous extract of fruits was prepared by maceration according to the method described by Pinheiro et al. (2020) with a slight modification. Briefly, 100 g of the lyophilized powder is homogenized with 1,000 ml of distilled water under magnetic stirring at room temperature for 24 h. The extract was filtered through a Whatman No. 4 filter paper, lyophilized, and then stored in the dark at −20°C until use (Ait Lhaj et al., 2021b).

### 2.2. HPLC-PDA-MS/MS analysis

A SHIMADZU LC-MS 8050 (Shimadzu, Japan, USA) LC system equipped with a triple quadrupole spectrometer and an ESI source was used for the phytochemical analysis. The chromatographic and mass spectrometric conditions were similar to those previously described by Tawfeek et al. (2023).

### 2.3. Antioxidant activity

#### 2.3.1. DPPH radical scavenging assay

The capacity to scavenge diphenylpicrylhydrazyl radical (DPPH·) was monitored according to a method reported before (Ghareeb et al., 2018). Briefly, 200 μl of AU extract (5.25–105 μg/ml) was added to 1.8 ml of a DPPH (0.2 mM) methanolic solution. The mixture was shaken vigorously and left standing at room temperature for 30 min in the dark. The reduction of the DPPH radical was measured at 517 nm vs. a blank solution by using a UV/Vis spectrometer. The DPPH· scavenging activity was calculated by using the equation:


$$\begin{array}{l} \text{\% Scavenging effect }=\;100\;\times \;\left({\text{A}}_{0}\;-{\text{A}}_{1}\right)/{\text{A}}_{0,} \end{array}$$

where A_0_ is the absorbance value of the DPPH blank sample and A_1_ is the absorbance value of the test solution.

#### 2.3.2. ABTS cation radical scavenging assay

The scavenging activity of AU extract against ABTS [2,2′-azino-bis(3-ethylbenzothiazoline-6-sulfonic acid)] radicals was monitored according to the method described by Arnao et al. (2001). Briefly, 200 μl of AU extract (5.25–105 μg/ml) was added to 2 ml of the ABTS^+^ working solution, prepared with ABTS (7 mM) and potassium persulfate (2.45 mM), incubated, and diluted with ethanol to achieve an absorbance of 0.70 ± 0.02 at 734 nm before analysis. The mixture was dark-incubated for 30 min at room temperature, and the absorbance was measured at 734 nm compared to a blank solution by using a UV/Vis spectrometer. The ABTS^+^ scavenging activity was calculated by using the following equation:


$$\begin{array}{l} \text{\% Scavenging effect }=\;100\;\times \;\left({\text{A}}_{0}\;{\text{A}}_{1}\right)/{\text{A}}_{0}, \end{array}$$

where A_0_ is the absorbance value of the ABTS^+^ blank sample and A_1_ is the absorbance value of the reaction mixture.

The antioxidant activity of freshly prepared aqueous solutions of ascorbic acid (vitamin C, within a range of 0.5–10 μg/ml) was also evaluated against DPPH and ABTS-free radicals. It served as a reference antioxidant for comparing the antioxidant activity of AU extract.

### 2.4. Biological evaluation

#### 2.4.1. Experimental approach

##### 2.4.1.1. Cell line

To investigate whether PQ may affect specifically the dopaminergic neurons by inducing mitochondrial metabolic alterations, we evaluated its effect on an established N27 rat dopaminergic neural cell line (SCC048 Sigma-Aldrich).

##### 2.4.1.2. Animals

Female and male Wistar rats were sourced from the animal facility at the University of Mohammed V in Rabat. They were housed in a controlled environment with a 12-h light/dark cycle and provided with standard food pellets and unlimited access to water. All experimental procedures were conducted following the guidelines and approval of the University of Mohammed V Animal Ethics Research Committee under the ethical approval number MAM5 70-8647.

For the study, female rats weighing between 180 and 200 g were paired with male rats at a ratio of 2:1. Each morning, the female rats were examined for the presence of vaginal plugs, which indicated successful mating and marked the beginning of gestational day 1 (GND 1). In cases where no vaginal plug was found, vaginal smears were collected and examined for the presence of sperm, thus confirming pregnancy.

##### 2.4.1.3. PQ treatment

A liquid commercial form of PQ Gramoxone (Syngenta Crop Protection, USA) was utilized. This formulation contained a PQ concentration of 200 g/l (Fernandes et al., 2021).

**Figure 1:**
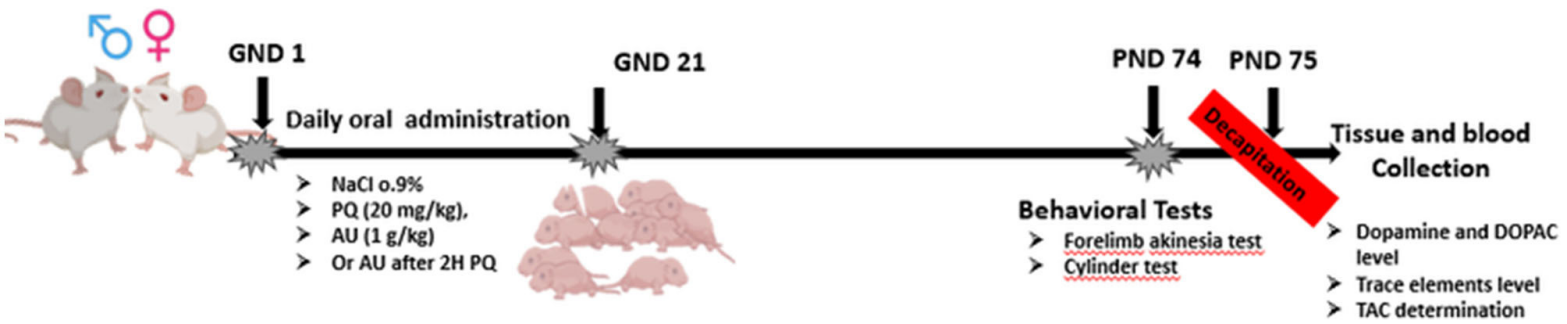
Timeline of the experimental protocol.

A total of 32 pregnant females were included in the study, with each group consisting of 8 females. They were administered different substances orally through gavage. The substances administered were saline, PQ at a dosage of 20 mg/kg, or AU at a dosage of 1 g/kg. This administration was performed once a day throughout the entire pregnancy period (21 days) from GND 1 to GND 21 according to the following protocol illustrated in Figure 1:

- The control group (Sal/Sal) received saline solution.
- The PQ group (PQ/Sal) was orally administered with PQ dichloride prepared in saline.
- The PQ/AU group was orally administered with AU 2 h after the oral administration of PQ.
- The Sal/AU group was orally administered with AU prepared in saline.

Following birth, pups remained with their dams until the post-natal day 21 (PND 21) after which they were culled to female offspring only.

#### 2.4.2. Cell viability test by MTT assay

The N27 rat dopaminergic neural cells viability was assessed using 3-(4,5-dimethyl-2-thiazolyl)-2,5-diphenyl-2H-tetrazolium bromide (MTT). The dopaminergic cells were plated in 96 well plates at a density of 4 × 104 cells per well and treated for 24 h with saline 0.9%, PQ (10, 100, 250, and 500 μM), AU (10, 100, 1,000, 5,000, and 10,000 μg/ml), or PQ/AU combination at 250 μM and 1,000 μg/ml or 5,000, respectively. Cultures were sustained in an incubator at 37°C for 4 h in the medium containing 0.5 mg/ml MTT. After washing with PBS, absorbance was measured at 490 nm using the Molecular Devices FlexStation II microplate reader.

#### 2.4.3. Mitochondrial metabolic analysis

The cells were maintained at 37°C and 5% CO_2_ on petri dishes in high glucose DMEM supplemented with 10% FCS, 3.75 mM glutamine, and 2.5 μg/ml fungizone (all Invitrogen). The dishes with the highest density of confluent cells were expanded (1:5), and their mitochondrial respiratory activity was assessed using the Mito stress test.

The metabolic rate of cultured N27 rat dopaminergic neural cells was determined by oxygen consumption rate (OCR) as a measure of mitochondrial function using the Seahorse Metabolic Bioanalyzer XFe24 (Agilent, UK). Oxidative metabolism of cells treated for 4 h with saline, PQ (250 μM), AU (5,000 μg/ml), or both was determined using the Mito stress test as per the manufacturer's instructions (Agilent). Briefly, the cells were stimulated by oligomycin (0.5 μM), carbonyl cyanide-4 (trifluoromethoxy) phenylhydrazone and oxidative phosphorylation uncoupler (FCCP; 1 μM), and rotenone (R) with antimycin A (A), a respiratory complex III inhibitor (R/A; 0.5 μM). All the reagents were dissolved in the Seahorse XF DMEM medium (pH 7.4; Agilent) supplemented with L-glutamine (2 mM), glucose (2.5 mM), and sodium pyruvate (2.5 mM). The mitochondrial respiratory parameters, including basal respiration, ATP production, proton leak, maximal respiration, spare capacity, and coupling efficiency were calculated and evaluated according to the manufacturer's recommendations.

#### 2.4.4. Behavioral analysis for the progeny rats

##### 2.4.4.1. The forelimb akinesia test

The test used to assess motor initiation, known as the forelimb akinesia test, was conducted according to previously described methods (Bonaccorso Marinelli et al., 2017). In summary, the rats were suspended in mid-air, allowing their body weight to rest on the forelimb being tested. Subsequently, each rat was propelled forward on an uneven surface, and the length of the adjusting step taken by the forelimb was measured using an adjacent ruler. Three trials were conducted for each forelimb, and an average step length was calculated for each limb.

##### 2.4.4.2. The limb-use asymmetry test

The limb-use asymmetry (cylinder) test, which evaluates the percentage of forelimb usage during exploratory behavior, was performed as described in studies (Meredith and Kang, 2006; Mabandla and Russell, 2010). Each animal was placed inside a transparent plexiglass cylinder measuring 20 cm in diameter and 30 cm in height for 5 min. The animal's behavior was recorded on video and later analyzed to determine the extent of wall exploration, contact with the wall, and landing after wall contact for both forelimbs. The percentage of limb use was then calculated using the following formula:


$$\begin{array}{l} \;\%\text{ Forelimb use =}\\ \frac{\text{number of contacts with contralateral limb+1/2 both}}{\text{ipsilateral limb use+ contralateral limb use+ both}}\text{X100}. \end{array}$$

#### 2.4.5. Tissue and blood collection

The offspring rats were euthanized through decapitation on PND 75, and blood samples were collected from their trunks into EDTA tubes. Blood plasma was acquired for the evaluation of total antioxidant capacity (TAC), and the striatal tissue was procured to quantify trace elements, dopamine, and DOPAC. The collection procedure adhered to sterile conditions and employed plastic utensils to avert potential metal contamination from dissection tools. Afterward, the tissue was delicately dried on the filter paper, weighed, and preserved at −20°C until subsequent analysis. Both blood plasma and striatal tissue were stored in a −80°C freezer until biochemical assays were conducted.

##### 2.4.5.1. Measurement of dopamine and DOPAC levels

In this study, we employed an HPLC system (Agilent Technologies HP 1100 series) equipped with electrochemical detection (Agilent Hewlett Packard 1049A) set to a potential of 750 mV. The analysis focused on dopamine and its metabolites, specifically 3,4-hydroxyphenylacetic acid (DOPAC). To prepare the samples, the striatal homogenate was thawed and then subjected to centrifugation at 4°C for 2 min at 12,000 g. For each sample, 10 μL of the resulting supernatant was injected into an Agilent LiChrospher 100 cartridge column (RP-18, 5 μm, 3 × 125 mm) maintained at 4°C.

The mobile phase composition consisted of 0.1 M Na2HPO4 (pH 3.3), 0.15 mM EDTA, and 25% methanol. A flow rate of 0.8 ml/min was used during the chromatographic separation. To identify the analytes (dopamine and DOPAC), their retention times were compared to those of corresponding standards. The concentration of each analyte was determined by calculating the area under the curve using the equation y = mx + c and expressed in ng/mg protein (Abboussi et al., 2020).

##### 2.4.5.2. Trace element analysis

The analysis of trace elements was conducted using inductively coupled plasma optical emission spectrometry (ICP-OES) based on a modified method described by Golden et al. (2001). In brief, the striatal tissue was sonicated in 2N hydrochloric acid (HCl) (0.5 ml) for homogenization. Subsequently, each sample was incubated with 70% perchloric acid (0.1 ml) at 50°C in a water bath for 24–36 h. After the incubation period, the samples were centrifuged at 3,500 rpm for 1 h and filtered through a 0.45 μm pore size filter using a filter syringe. The analysis of samples and standards was performed using the Perkin Elmer Optima 5,300 DV Optimal Emission Spectrometer (Waltham MA, USA).

##### 2.4.5.3. Total antioxidant capacity

The total antioxidant capacity (TAC) is a measure of the collective antioxidant activity displayed by biomolecules in a sample. To assess TAC, whole blood was subjected to centrifugation at 3,500 rpm for 10 min, resulting in the collection of plasma. The plasma was then analyzed for TAC using the OxiSelect™ Total Antioxidant Capacity Assay kit (Cell Biolabs Inc., UK) following the instructions provided by the manufacturer.

### 2.5. Statistical analysis

Statistical analysis was performed using Graph-Pad Prism software 8.0. Statistical significance was set at a *p*-value of <0.05, and data are expressed as mean ± SEM. The normality of data was assessed by the Shapiro test. Differences in mean values were analyzed with a one-way or two-way ANOVA test followed by a Bonferroni *post-hoc* test for all experimental data. All figures were prepared using Graph-Pad Prism version 8.0.

## 3. Results

### 3.1. Chemical composition of AU extract

**Figure 2:**
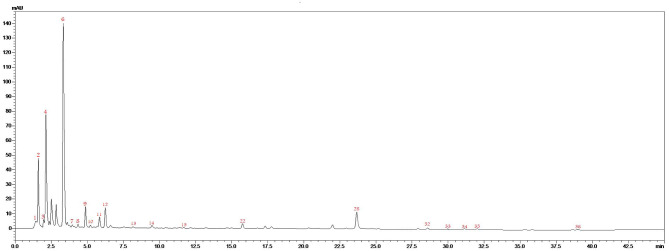
LC/MS/MS chromatogram of the aqueous extract from *A. unedo* fruit.

**Figure 3:**
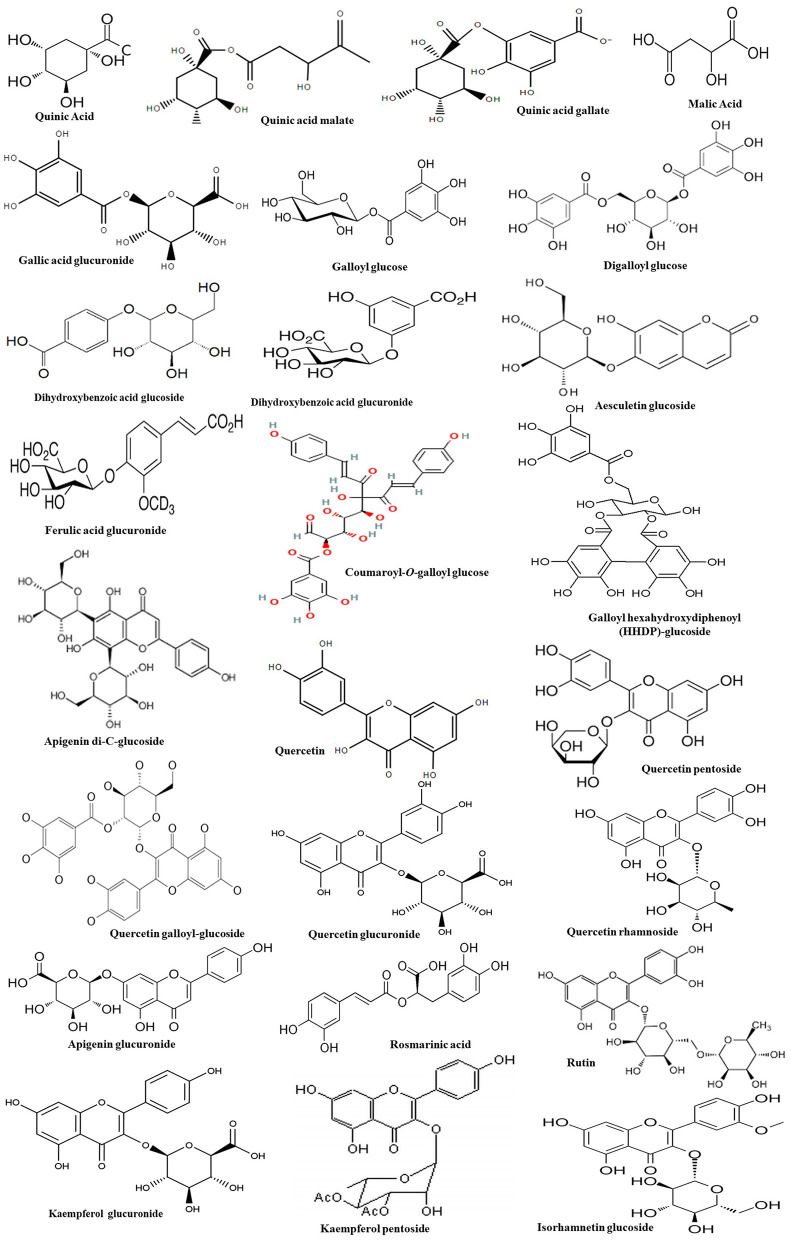
Chemical structures of the bioactive compounds found in the aqueous extract from *A. unedo* fruit.

Figure 2 and Table 1 represent the chromatogram and the phytoconstituents of the aqueous extract from *A. unedo* fruits, respectively. Phytochemical analysis of the aqueous extract from *A. unedo* fruits, *via* LC-MS/MS, revealed 35 compounds, mainly phenolic and organic acids, and flavonoids, and their chemical structures are illustrated in Figure 3. Quinic acid, an example of organic acid, along with its malate, gallate, and digallate derivatives were tentatively identified based on their molecular weights (191, 307, 343, and 495, respectively) and corresponding fragment 191 (Table 1). Several gallic acid derivatives, examples of phenolic acid, were also present. These include galloyl glucose, gallic acid- malate, -shikimate, -glucuronide, and -rhamnosyl gallate along with two digalloyl glucose isomers (Table 1). Noteworthy, galloyl hexahydroxydiphenoyl (HHDP)-glucoside, an example of ellagitannins, was also detected in the extract. Mono- and di-glycosides of apigenin, kaempferol, quercetin, and isorhamnetin, examples of flavonoid glycosides, were found in the extract as well.

**Table 1:** Annotated metabolites from the aqueous extract from *A. unedo* fruits.

**N°**	**Rt (min)**	**[M-H]^−^**	**Mass fragments**	**Proposed bioactive compounds**
1	1.46	191	108	Quinic acid
2	1.64	133	115	Malic acid
3	1.94	307	191	Quinic acid malate
4	2.53	331	169	Galloyl glucose
5	3.25	343	191	Quinic acid gallate
6	3.44	331	169	Galloyl glucose
7	4.10	315	153	Dihydroxybenzoic acid glucoside
8	4.34	345	168	Gallic acid glucuronide
9	4.82	331	169	Galloyl glucose
10	5.18	329	153	Dihydroxybenzoic acid glucuronide
11	5.42	325	169	Gallic acid shikimate
12	6.21	315	153	Dihydroxybenzoic acid glucoside
13	8.66	339	177	Aesculetin glucoside
14	9.51	495	191	Quinic acid digallate
15	11.67	483	169	Digalloylglucose
16	12.29	453	169	Pyrogallol methyl galloyl glucose
17	13.17	483	169	Digalloylglucose
18	13.42	369	193	Ferulic acid glucuronide
19	13.95	359	197	Synergic acid glucoside
20	14.80	577	169	Coumaroyl-*O*-galloyl glucose
21	15.58	467	169	Gallic acid rhamnosyl-gallate
22	15.88	633	301	Galloyl hexahydroxydiphenoyl (HHDP)-glucoside
23	16.73	593	353	Apigenin di-*C*-glucoside
24	19.91	477	169	Coumaroyl-O-galloyl glucose
25	22.85	615	301	Quercetin galloyl-glucoside
26	22.92	433	301	Quercetin pentoside
27	23.08	477	315	Isorhamnetin glucoside
28	23.86	609	301	Rutin
29	24.66	477	301	Quercetin glucuronide
30	24.09	461	285	Kaempferol glucuronide
31	27.90	433	301	Quercetin pentoside
32	28.32	447	301	Quercetin rhamnoside
33	29.77	445	269	Apigenin glucuronide
34	31.03	359	197	Rosmarinic acid
35	31.88	417	285	Kaempferol pentoside
36	38.96	301	151	Quercetin

### 3.2. *In vitro* antioxidant capacity of AU extract

In the current study, the antioxidant activity of AU extract was estimated through DPPH and ABTS assay. AU extract and vitamin C displayed promising antioxidant activities in a dose-dependent manner. AU extract displayed stronger *in vitro* radical scavenging activity against DPPH radicals than ABTS radicals (Table 2).

**Table 2:** Antioxidant potential of the AU extract compared to the positive control vitamin C.

**Samples**	**DPPH (IC_50_, μg/mL)**	**ABTS (IC_50_, μg/mL)**
AU extract	5.09 ± 0.22	11.55 ± 0.16
Vitamin C	2.80 ± 0.03	5.90 ± 0.18

Results are expressed as mean ± SEM (n = 3).

### 3.3. Effect of AU extract on PQ-decreased dopaminergic cells viability

**Figure 4:**
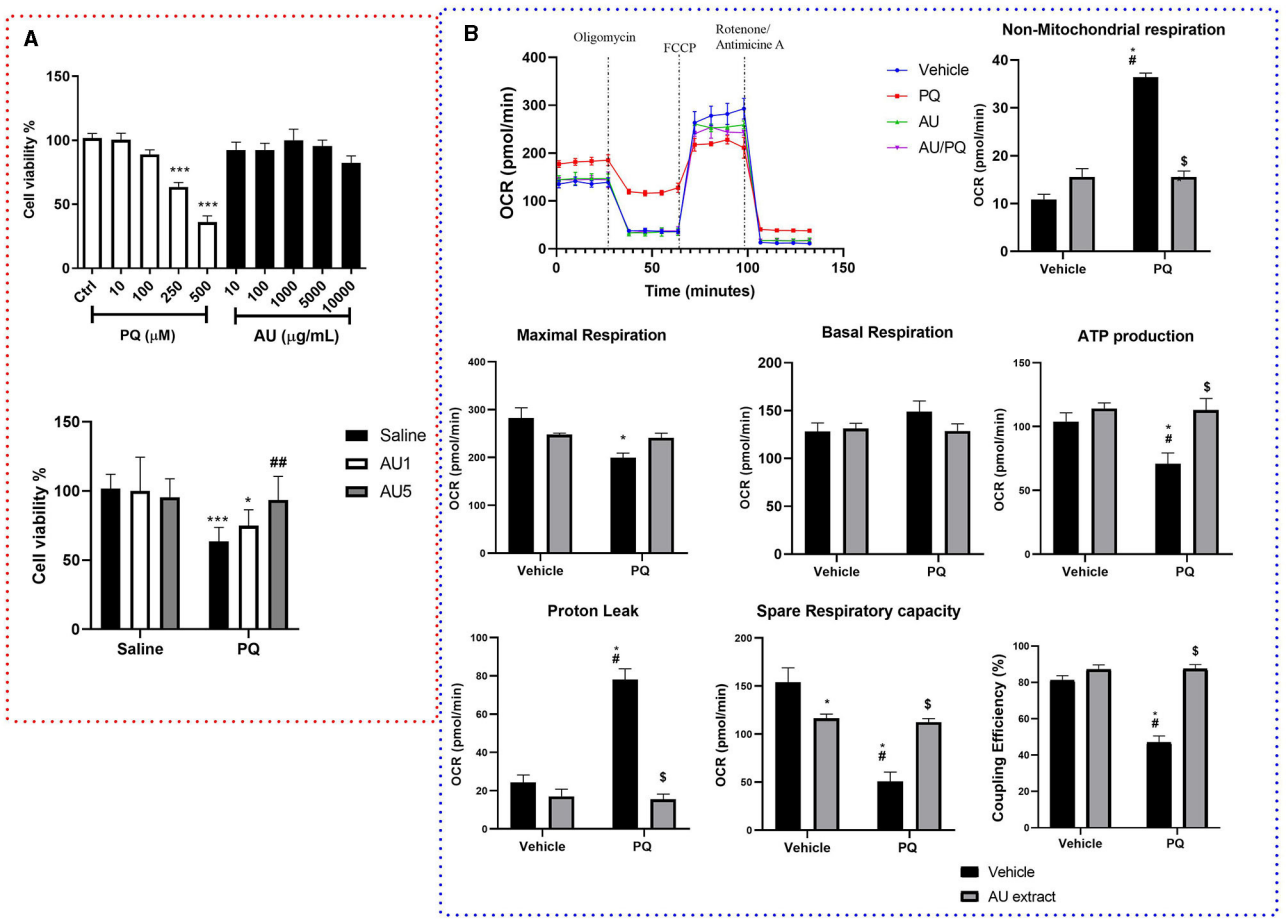
**(A)** Results of MTT assay on dopaminergic cell viability after 4 h of exposure to different concentrations of PQ (10–500 μM), AU (10–10,000 μg/mL) (upper graph), and the combination of PQ (250 μM) and AU at 1,000 μg/ml (AU1) and 5,000 μg/ml (AU5) (lower graph). Data were normalized as a percentage of control (complete culture media). Results are expressed as mean ± SEM from eight biological replicates (upper graph). Ordinary one-way ANOVA multiple comparisons test significant differences, ***p < 0.001 compared with the control group (lower graph). Bonferroni's multiple comparisons test revealed significant differences; *p < 0.05 and ***p < 0.001 compared to all saline groups, ^##^*p* < 0.01 compared to the PQ group. **(B)** AU extract (5,000 μg/ml) and PQ (250 μM) effects on dopaminergic cells mitochondrial respiratory parameters. Data are presented as the mean of five independent experiments, as means ± SEM, significance was assessed by the Bonferroni *post-hoc* test; *p < 0.05 vs. Vehicle: Vehicle, ^#^*p* < 0.05 vs. Vehicle: AU, ^$^*p* < 0.05 vs. PQ: Vehicle.

As depicted in Figure 4A, MTT results showed that PQ-affected dopaminergic cells viability where the stimulation with PQ concentrations of 250 and 500 μM significantly decreased cell viability compared to the control group (*p* < 0.0001), while the treatment with AU extract on different concentrations seems to have no significant effect on cells viability (*p* > 0.05). The AU extract at 5,000 μM significantly enhanced cell viability affected by PQ, while the AU extract at 1,000 μM seems to have no significant effect against PQ-induced cytotoxicity (*p* > 0.9999).

### 3.4. AU extract effects on PQ-induced mitochondrial bioenergetics impairment

XFe24 Seahorse analyzer was used to determine whether PQ-induced bioenergetic mitochondrial profile impairment in N27 dopaminergic neural cells is alleviated by the AU extract. Oxidative respiration expressed as oxygen consumption rate (OCR) (Figure 4B) was measured for neural cells after 4 h of PQ, AU, or both exposures. Two-way ANOVA analysis of Mito stress test data revealed significant interactions between PQ and AU treatments on maximal respiration [*F*_(1, 16)_ = 8.67, *p* = 0.0095], non-mitochondrial respiration [*F*_(1, 16)_ = 103.3, *p* < 0.0001], proton leak [*F*_(1, 16)_ = 44.67, *p* < 0.0001], spare respiratory capacity [*F*_(1, 16)_ = 27.97, *p* < 0.0001], ATP production [*F*_(1, 16)_ = 4.678, *p* = 0.0460], and ATP coupling efficiency [*F*_(1, 16)_ = 39.29, *p* < 0.0001]. While no significant effect was shown on basal respiration rate [*F*_(1, 16)_ = 1.86, *p* = 0.1912]. The Bonferroni *post-hoc* test showed that PQ significantly increased the non-mitochondrial respiration and the proton leak compared to the control (Vehicle: Vehicle, *p* < 0.0001 and *p* < 0.0001, respectively), AU-treated cells (Vehicle: AU, *p* < 0.0001 and *p* < 0.0001, respectively), and AU-treated cell after PQ exposure (PQ: AU, *p* < 0.0001 and *p* < 0.0001). It significantly decreased the maximal respiration rate compared to the control group (*p* = 0.0020). In addition, PQ significantly decreased the spare respiratory capacity of neuronal cells, their ATP production, and their coupling efficiency compared to all vehicle groups, and the AU extract inhibits this side effect by increasing significantly rate of these parameters (*p* = 0.0016, *p* = 0.0056, and *p* < 0.0001, respectively; PQ: vehicle vs. PQ: AU).

### 3.5. Behavioral analysis

#### 3.5.1. Effect of PQ and the AU extract on the forelimb stepping test

**Figure 5:**
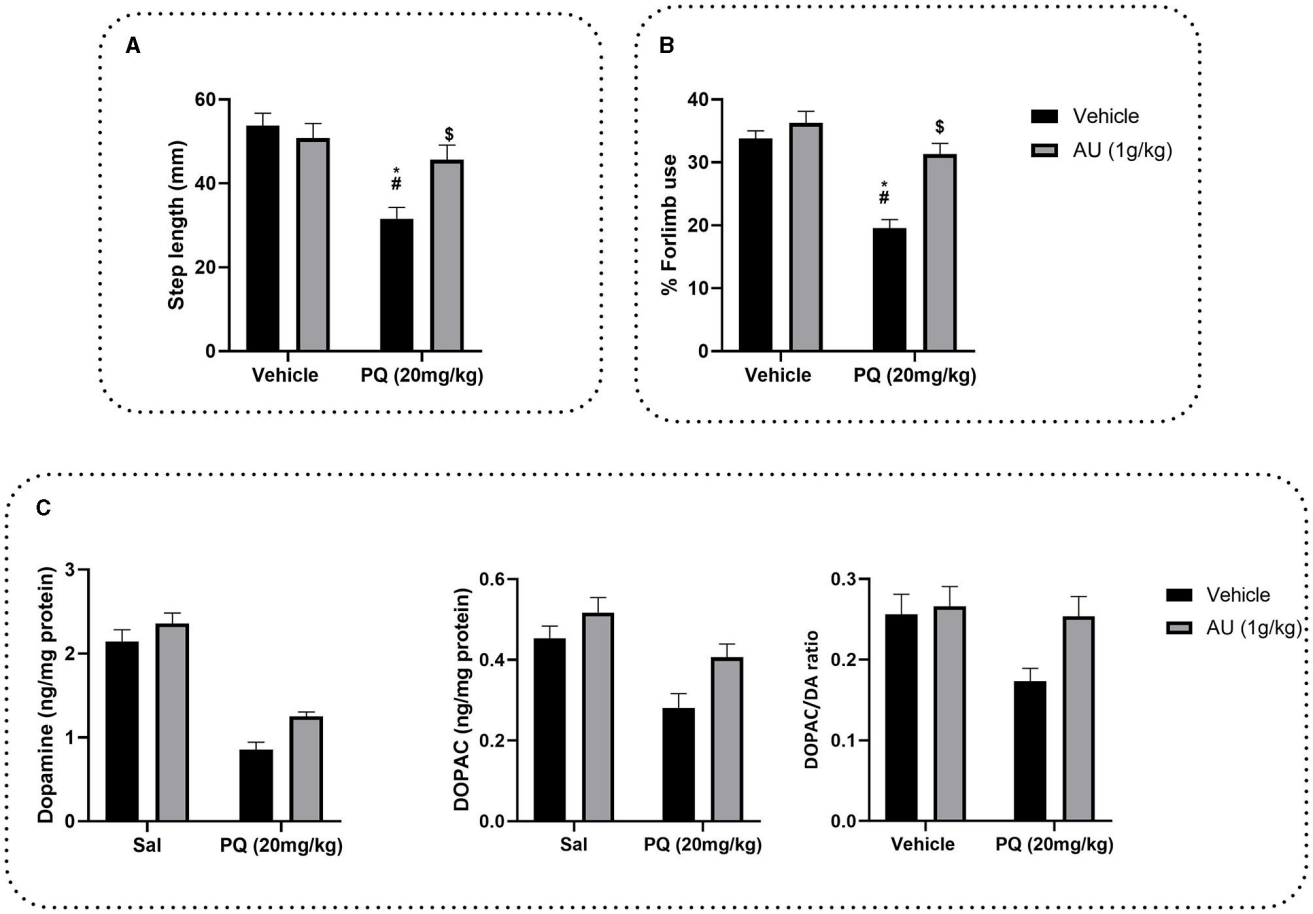
**(A)** Effects of the AU extract on PQ-reduced step length in the forelimb stepping test. **(B)** Effects of the AU extract on PQ induced a deficit in the usage of the contralateral forelimbs by rats' progeny in the cylinder test for asymmetry. **(C)** Effects of prenatal PQ exposure and treatment with AU on dopamine (DA), DOPAC levels in the striatum of rats' progeny, and DA turnover expressed as DOPAC/DA ratio. Each value is expressed as mean ± SEM (*n* = 8). Significance was assessed by the Bonferroni *post-hoc* test when there is significant interaction between the AU extract and PQ treatment; *p < 0.05 vs. Vehicle: Vehicle, ^#^*p* < 0.05 vs. Vehicle: AU, ^$^*p* < 0.05 vs. PQ: Vehicle.

To evaluate the AU extract effect on PQ-induced akinesia on rat progeniture, the step length was estimated by the forelimb stepping test. Two-way ANOVA demonstrated a significant interaction between the group treated with the AU extract after the pretreatment with PQ and the group treated with PQ alone [*F*_(1, 28)_ = 7.112, *p* = 0.0126]. As depicted in Figure 5A, rats treated with the AU extract alone showed no significant effect on the step length of their progeniture compared to the vehicle group (*p* > 0.9999). Posttreatment of rats with the AU extract at a dose of 1 g/kg after PQ exposure significantly reversed akinesia (*p* = 0.0251) observed in rat progeny by improving their step length.

#### 3.5.2. AU treatment inhibits PQ-induced deficit in contralateral forelimb use

To test the effect of AU treatment on PQ prenatally exposure induced a deficit in motor coordination, and contralateral forelimb use was evaluated by the cylinder test for asymmetry (Figure 5B). Two-way ANOVA analysis revealed a significant interaction between PQ exposure and AU treatment [*F*_(1, 28)_ = 8.944, *p* = 0.0057]. As depicted in Figure 5B, Bonferroni's multiple comparison test revealed that prenatal exposure to PQ induced a significant deficit in the usage of the contralateral forelimb by rat progeniture (*p* < 0.0001). This deficit was significantly decreased by AU treatment (*p* < 0.0001).

#### 3.5.3. AU treatment reduces PQ-induced dopamine depletion in the striatum

The DA and DOPAC levels measured in the striatum indicated that PQ decreased dopamine levels [*F*_(1, 28)_ = 125.4, *p* < 0.0001] and its metabolite DOPAC [*F*_(1, 28)_ = 17.63, *p* = 0.0002] as well as DA turnover [*F*_(1, 28)_ = 4.364, *p* = 0.0459] (Figure 5C). However, AU increased significantly both DA [*F*_(1, 28)_ = 8.188, *p* = 0.0079] and DOPAC [*F*_(1, 28)_ = 7.799, *p* = 0.0093] levels as revealed by ANOVA two-way analysis. No interaction between PQ and AU treatments was revealed when evaluating the DA level [*F*_(1, 28)_ = 0.7071, *p* = 0.4075], neither the DOPAC level [*F*_(1, 28)_ = 0.8213, *p* = 0.3725] nor DA turnover [*F*_(1, 28)_ = 2.365, *p* = 0.1353].

#### 3.5.4. PQ and AU effects on striatum trace elements level

**Figure 6:**
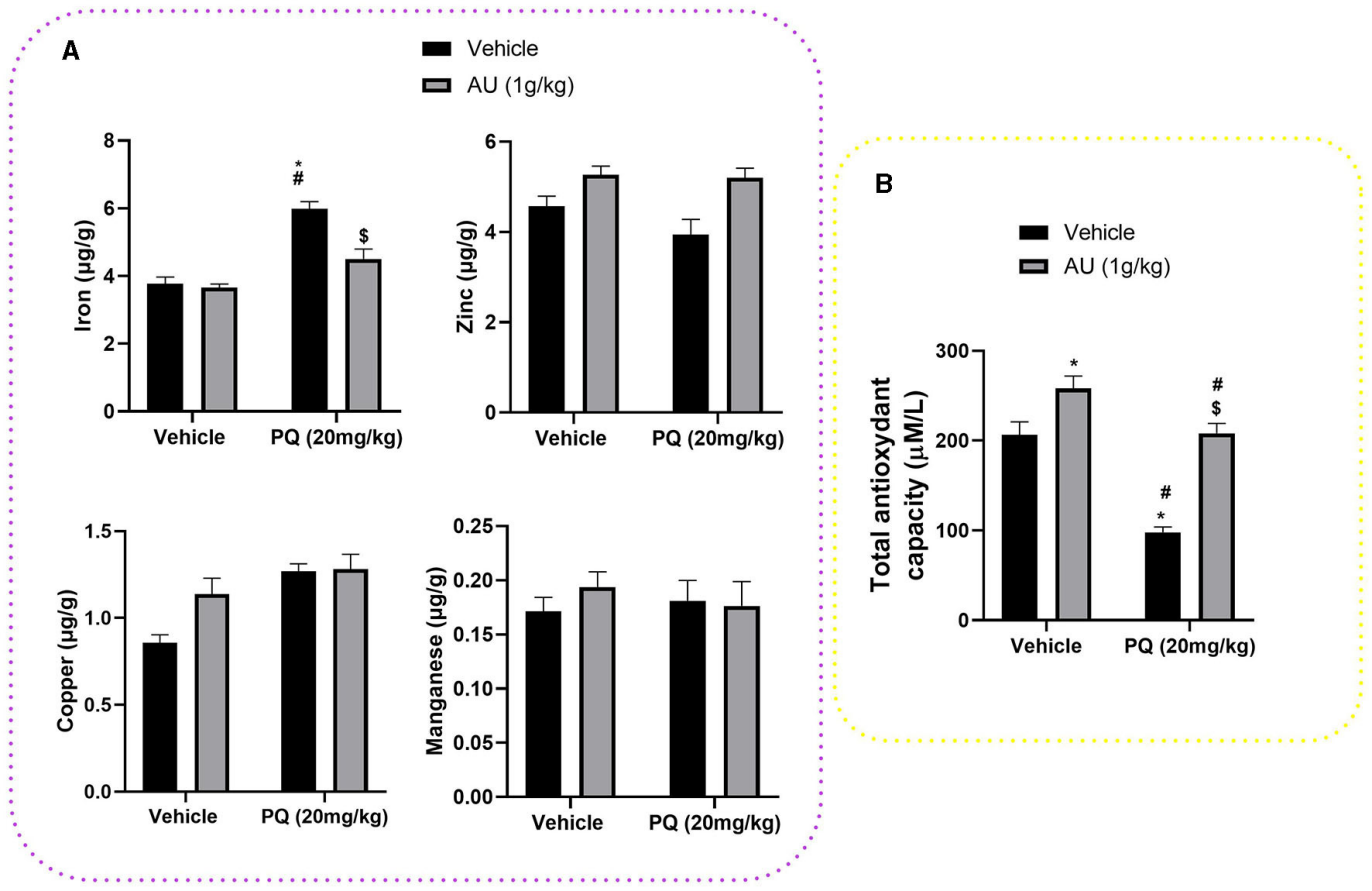
**(A)** Trace element levels at the striatal tissue of PND 75 prenatally exposed to the PQ (20 mg/kg) and the effect of the co-treatment with the AU extract (1 g/kg). **(B)** PQ prenatal exposure decreased TAC in the blood plasma of rats at PND 75 and treatment with AU extract alleviated this effect. Data are expressed as mean ± SEM (*n* = 8). Significance was assessed by the Bonferroni *post-hoc* test when there is significant interaction between AU extract and PQ treatments; *p < 0.05 vs. Vehicle: Vehicle, ^#^*p* < 0.05 vs. Vehicle: AU, ^$^*p* < 0.05 vs. PQ: Vehicle.

The effect of the prenatal exposure of PQ on iron, copper, zinc, and manganese levels at the striatal tissue of PND 75 is shown in Figure 6A. Two-way ANOVA revealed significant interactions between prenatal PQ exposure and AU treatment [*F*_(1, 28)_ = 10.66, *p* = 0.0029] on iron levels, where PQ significantly increases its level in the striatal tissue of rats at the PND75 compared to the control group (Vehicle: Vehicle; *p* < 0.0001) and the group prenatally exposed to the AU extract (Vehicle: AU; *p* < 0.0001). The Bonferroni *post-hoc* test also showed that this effect is significantly alleviated with co-treatment with the AU extract alongside PQ exposure (PQ: AU; *p* = 0.0002). However, no significant interactions were shown on striatal levels of zinc [*F*_(1, 28)_ = 1.330; *p* = 0.2585], copper [*F*_(1, 28)_ = 3.499; *p* = 0.0719], and manganese [*F*_(1, 28)_ = 0.6196; *p* = 0.4378].

#### 3.5.5. AU treatment hides PQ prenatal exposure-induced TAC decrease

As depicted in Figure 6B, a significant effect of AU and PQ on TAC in the blood plasma of rats at PND 75 is shown. Two-way ANOVA revealed significant interactions between AU and PQ treatments [*F*_(1, 28)_ = 6.265; *p* = 0.0184], where PQ prenatal exposure reduced significantly the TAC in the blood plasma compared to the control group (Vehicle: Vehicle; *p* < 0.0001), while the co-treatment with AU extract significantly hides this PQ effect by increasing TAC in the blood plasma (PQ: AU, *p* < 0.0001). In addition, AU prenatal treatment significantly improved the TAC in the plasma blood of rats at PND75 compared to the control group (Vehicle: Vehicle, *p* = 0.0264), which highlights the antioxidant effect of this extract related to its bioactive compounds.

## 4. Discussion

PQ, a well-known herbicide for its irreversibly detrimental effects on the brain in animal models, causes neuronal cell death in the substantia nigra (Liou et al., 1996; Manning-Bog et al., 2002; Somayajulu-Nitu et al., 2009), behavioral abnormalities (Fernagut et al., 2007), oxidative stress (Chen et al., 2010; Ishola et al., 2019), and mitochondrial respiration impairments (Czerniczyniec et al., 2015; Wang et al., 2018; Ishola et al., 2019). Recently, it was demonstrated that prenatal PQ exposure induces neurotoxicity in mouse offspring, leading to several neurochemical alterations and altered brain development (Ait-Bali et al., 2016; Hamdaoui et al., 2022). In this regard, treatments for PQ poisoning may include the modulation of the levels of glutathione-utilizing antioxidant agents, selenium compounds, as well as metal chelators utilization (Blanco-Ayala et al., 2014). However, some of these are not long-lasting and may show side effects.

In this respect, numerous studies highlighted that phytoconstituents, including polyphenols, may exert antioxidant activities. As reported in Table 1, in our study, we used an aqueous AU extract that primarily comprises phenolic compounds alongside flavonoid glycosides, which are widely known for their antioxidant activities (Grabska-Kobylecka et al., 2020). Based on these findings, we first assessed the neuroprotective effects of AU extract against PQ-induced cytotoxicity and mitochondrial impairments in the N27 dopaminergic cell line. Then, we investigated the effect of AU extract on motor function, dopamine, and trace element levels in the striatum as well as its effect on plasmatic total antioxidant capacity (TAC) in PQ-prenatally exposed Wistar rats.

For that, the rats were treated with the AU extract 2 h after the oral administration of PQ to minimize any potential interactions between the two treatments. This decision was based on a previous study investigating the toxicokinetics and bioavailability of PQ. The study reported a terminal half-life of [14C] PQ of 68.0 ± 23.0 min following intragastric administration, indicating a rapid clearance from the bloodstream (Chui et al., 1988). By allowing a 2-h interval between the two treatments, we aimed to ensure that the effects of PQ administration were adequately established before introducing the AU extract. This approach was employed to enable a focused evaluation of the neuroprotective effects of the AU extract without any confounding influences from immediate interactions between the two compounds.

**Figure 7:**
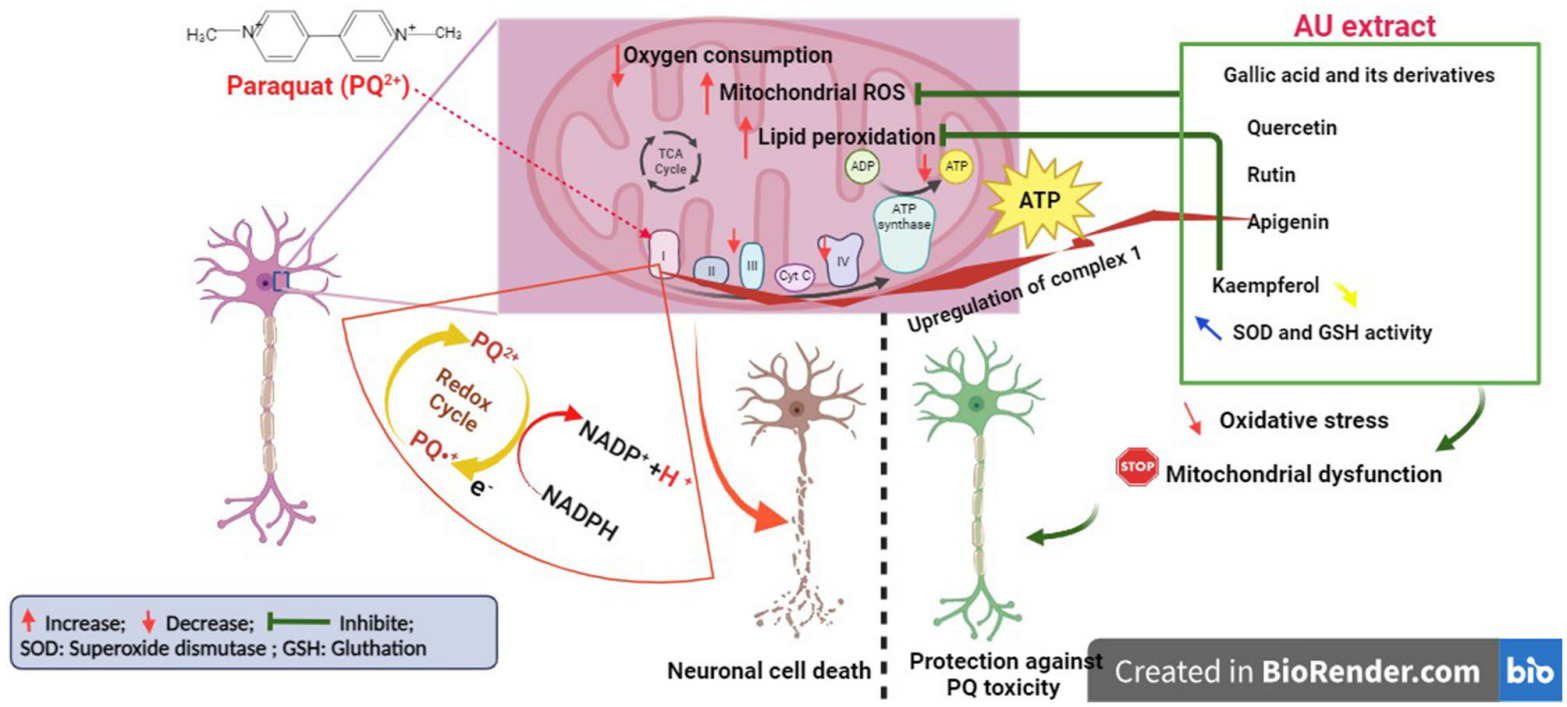
Illustration of the mechanism behind the antioxidant capabilities of AU extract protecting neurons from PQ-induced cell death (Created with BioRender.com).

Our results demonstrated that PQ treatment at 250 and 500 μM significantly decreased cell viability, confirming the findings of earlier studies on PQ neurotoxicity (Chen et al., 2008; Ortiz-Ortiz et al., 2009; Han et al., 2019). These studies have shown that PQ-induced dopaminergic cell death might be mediated by its effect on dopaminergic neurons (Choi et al., 2008; See et al., 2022). They demonstrated that PQ is recycled *via* mitochondrial complex I, leading to its activation and ROS production, which causes oxidative stress and apoptosis. Treatment of N27 cells with the AU extract at 5,000 μg/mL significantly protected them against PQ toxicity. This effect may be principally mediated by the presence of several antioxidant compounds, as reported before, particularly gallic acid and its derivatives, quercetin, apigenin, kaempferol, and rutin (Figure 7). Gallic acid and its derivatives have a potential protective effect against neuronal damage by inhibiting mitochondrial apoptotic signaling molecules and scavenging ROS, including hydroxyl radicals, superoxide anions, and hydrogen peroxide (Shabani et al., 2020). Furthermore, there is substantial scientific evidence documenting the ability of flavonoids, specifically quercetin and apigenin, to mitigate the loss of neuronal cells by reducing oxidative stress. Quercetin achieves this effect by attenuating the activities of xanthine oxidase and nitric oxide (NO) synthase and upregulating the activity of mitochondrial complex I (Karuppagounder et al., 2013; Islam et al., 2021). On the other hand, apigenin exerts its neuroprotective effects by inhibiting dopaminergic neuronal cell death through anti-inflammatory mechanisms mediated by the upregulation of peroxisome proliferator-activated receptor-gamma (PPARγ) and the suppression of oxidative stress induced by rotenone (Li et al., 2016; Anusha et al., 2017). Additionally, rutin and kaempferol have been reported to protect dopaminergic neurons against 6-OHDA and rotenone-induced neurotoxicity in SH-SY5Y cells and primary neurons, respectively. This neuroprotection is highlighted by increasing superoxide dismutase and glutathione activity and reducing lipid peroxidation levels (Filomeni et al., 2012; Moshahid Khan et al., 2012).

Our findings also demonstrated that PQ exposure at a low concentration (250 μM) led to impairments of mitochondrial respiration in N27 dopamine cells. This is consistent with other studies, collectively highlighting the detrimental effects of PQ on mitochondrial respiration in neuronal cells, mainly affecting complex I and IV activity, leading to impaired electron transport chain function and reduced ATP production (Czerniczyniec et al., 2011; Wang et al., 2018; de Oliveira Souza et al., 2019). In our study, the AU extract significantly mitigated the PQ-induced abnormal mitochondrial functioning of N27 dopamine cells. It was observed to increase oxygen consumption rate (OCR), decrease proton leak, and enhance the maximal respiratory capacity of dopaminergic neurons and their coupling efficiency. These effects could be mediated by its antioxidant properties, which can help counteract PQ-induced oxidative stress by scavenging reactive oxygen species (ROS), protecting mitochondrial components, and maintaining their functional integrity, thereby supporting normal mitochondrial respiration (Dabaghi-Barbosa et al., 2005; Wolf et al., 2010; Xiao et al., 2022). Additionally, it has been demonstrated that phenolic acids present in the AU extract activate Nrf2 (nuclear factor erythroid 2-related factor 2) (Tambe et al., 2023), which is associated with the activation of mitochondrial biogenesis and enhancement of mitochondrial activity by upregulating the expression of key genes involved in mitochondrial DNA replication and maintaining the integrity of the mitochondrial membrane potential, which is essential for mitochondrial function (Tufekci et al., 2011; Ryoo and Kwak, 2018).

Behavioral assessments revealed that prenatal exposure to PQ resulted in motor deficits resembling akinesia in rat offspring, consistent with previous studies indicating PQ-induced motor incoordination and reduced spontaneous motor behavior due to degeneration of the nigrostriatal dopaminergic system (Li et al., 2005; Colle et al., 2020). Interestingly, the administration of AU significantly mitigated these motor deficits caused by PQ, suggesting a positive effect on motor function. This beneficial effect could potentially be attributed to the presence of AU polyphenols, particularly gallic acid, which has been reported as an effective compound against PQ/iron co-treatment-induced impairment of normal movement functionality in *Drosophila melanogaster* (Jimenez-Del-Rio et al., 2010). Furthermore, the loss of dopaminergic neurons is associated with a decline in dopamine (DA) levels and its metabolite, DOPAC, leading to a reduction in the DOPAC/DA ratio (DA turnover). Our results demonstrated that AU alleviated PQ-induced dopamine depletion and decreased DA turnover in the striatal tissue of rat progeny. These effects could be mediated by the bioactive compounds of AU, particularly kaempferol, quercetin, and apigenin. Kaempferol, for instance, has been shown to prevent MPTP-induced Parkinson's disease in C57BL/6 mice by inhibiting dopaminergic neuron loss, increasing dopamine and DOPAC levels, and enhancing DA turnover. These effects were associated with the increased activity of antioxidant enzymes such as superoxide dismutase (SOD) and glutathione peroxidase (GPx), as well as a reduction in the levels of malondialdehyde (MDA), a marker of lipid peroxidation and oxidative damage. Additionally, kaempferol improved performance in spontaneous motor activity tests, indicating its positive impact on motor behavior (Shahpiri et al., 2016). Similarly, recent studies have demonstrated the neuroprotective effects of quercetin in attenuating rotenone-induced toxicity by reducing redox stress in the striatum, optimizing dopamine metabolism, and improving neuronal density depletion (Josiah et al., 2022). In the same Parkinson's disease model, apigenin has been shown to modulate dopaminergic neurotransmission by downregulating α-synuclein expression and enhancing dopamine biosynthesis through the upregulation of dopamine D2 receptor protein expression and tyrosine hydroxylase (Anusha et al., 2017).

Furthermore, our findings revealed that prenatal exposure to PQ disrupted iron homeostasis, leading to increased striatal iron levels. PQ has been shown to interfere with iron metabolism and its release from Fe-S cluster proteins in the mitochondrial respiratory chain, resulting in iron accumulation (Dixon and Stockwell, 2014; Onukwufor et al., 2022). This iron accumulation contributes to oxidative stress and neurodegeneration through the generation of reactive oxygen species (ROS) *via* the Fenton reaction. Interestingly, treatment with the AU extract effectively attenuated the PQ-induced increase in striatal iron levels, suggesting its potential as an iron-chelating agent. Previous studies have reported the iron-chelating capacity of flavonoids and other compounds present in the AU extract, highlighting their ability to sequester excessive iron and prevent iron-induced oxidative damage (Porfírio et al., 2014; Kejík et al., 2021). Importantly, PQ exposure did not significantly alter the levels of other metal ions, such as copper, zinc, and manganese, in the striatal tissue. This selectivity of PQ in inducing alterations specifically in iron homeostasis without affecting other metal ions is noteworthy. Similarly, treatment with the AU extract did not significantly affect the levels of these metals, indicating its specific action in addressing iron dysregulation associated with prenatal PQ exposure.

Additionally, our results demonstrated that prenatal PQ exposure resulted in a reduction in total antioxidant capacity (TAC) in the blood plasma of the rat offspring, indicating a diminished ability to neutralize ROS and protect against oxidative damage. This effect can be attributed to PQ-induced ROS generation, as previously reported, which depletes endogenous antioxidants in the body, including enzymes such as superoxide dismutase (SOD), catalase (CAT), and glutathione (GSH) peroxidase (Sengupta et al., 2017; Hasanuzzaman et al., 2018). These enzymes play a crucial role in scavenging and neutralizing ROS; however, PQ overwhelms their capacity, leading to decreased antioxidant levels. Notably, treatment with the AU extract effectively mitigated this effect by significantly improving TAC in the blood plasma of PQ-exposed rat offspring. This can be attributed to the bioactive compounds present in AU, particularly polyphenols and flavonoids, which have been shown to upregulate the activity and expression of SOD, CAT, and GSH while downregulating the expression of malondialdehyde (MDA), a marker of oxidative damage (Alvarez-Suarez et al., 2011; Gao et al., 2022). Consequently, the AU extract helps maintain a balance between ROS production and antioxidant defense in the body.

In summary, our results suggested that the AU extract inhibited prenatal PQ-induced neurotoxicity by preserving dopaminergic cell viability, improving mitochondrial function, ameliorating motor deficits, and restoring iron homeostasis. The polyphenolic compounds present in the AU extract, such as gallic acid, quercetin, apigenin, kaempferol, and rutin, likely contribute to these neuroprotective effects through their antioxidant, anti-apoptotic, and iron-chelating properties. Further investigations are warranted to elucidate the underlying mechanisms and assess the translational potential of AU extract as a therapeutic intervention for PQ poisoning and related neurodegenerative disorders.

## Data availability statement

The original contributions presented in the study are included in the article/Supplementary material, further inquiries can be directed to the corresponding authors.

## Ethics statement

The animal study was approved by University of Mohammed V Animal Ethics Research Committee, under the Ethical Approval Number MAM5 70-8647. The study was conducted in accordance with the local legislation and institutional requirements.

## Author contributions

OA and ZA developed the overall study objectives. OA, MS, KT, and MA designed the methodology. ZA, HI, SE, MS, and OA performed most of the experimental analysis and wrote the first draft of the manuscript. FA pre-processed experimental data and assisted in some experiments and manuscript writing. MS and OA assisted in collating the data and interpreting the results. WM, KT, MA, and OA reviewed and revised the manuscript. All authors contributed to the article and approved the submitted version.
