# Supplementary figures and images for: Bioactive strawberry fruit (Arbutus unedo L.) extract remedies paraquat-induced neurotoxicity in the offspring prenatally exposed rats
Source: Front Neurosci. 2023 Oct 12;17:1244603. doi: 10.3389/fnins.2023.1244603 (PMC10600521; doi:10.3389/fnins.2023.1244603)

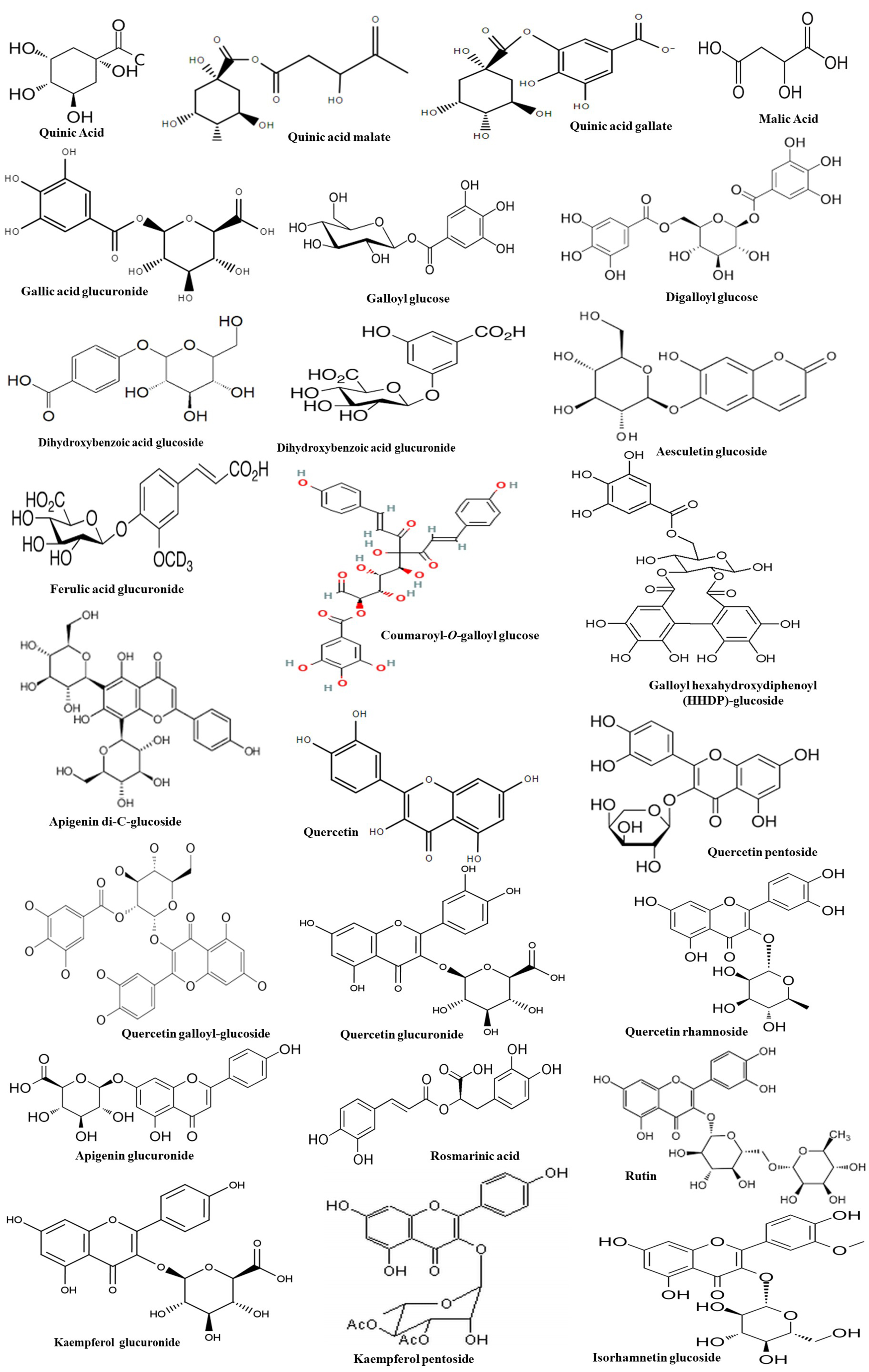

Supplement: Supplementary file 10 [file Image_1.JPEG]
